# Scenarios and methods that induce protruding or released CNTs after degradation of nanocomposite materials

**DOI:** 10.1007/s11051-013-1504-x

**Published:** 2013-03-06

**Authors:** Sabine Hirth, Lorenzo Cena, Gerhard Cox, Željko Tomović, Thomas Peters, Wendel Wohlleben

**Affiliations:** 1 BASF SE, 67056 Ludwigshafen, Germany; 2 CDC/NIOSH, Morgantown, WV 26505 USA; 3 BASF Polyurethanes GmbH, GMU/UE, Elastogranstrasse 60, 49448 Lemfoerde, Germany; 4 The University of Iowa, Iowa City, IA 52242 USA

**Keywords:** Nanocomposites, Degradation, Life-cycle, Characterization for toxicology purposes

## Abstract

**Abstract:**

Nanocomposite materials may be considered as a low-risk application of nanotechnology, if the nanofillers remain embedded throughout the life-cycle of the products in which they are embedded. We hypothesize that release of free CNTs occurs by a combination of mechanical stress and chemical degradation of the polymer matrix. We experimentally address limiting cases: Mechanically released fragments may show tubular protrusions on their surface. Here we identify these protrusions unambiguously as naked CNTs by chemically resolved microscopy and a suitable preparation protocol. By size-selective quantification of fragments we establish as a lower limit that at least 95 % of the CNTs remain embedded. Contrary to classical fiber composite approaches, we link this phenomenon to matrix materials with only a few percent elongation at break, predicting which materials should still cover their CNT nanofillers after machining. Protruding networks of CNTs remain after photochemical degradation of the matrix, and we show that it takes the worst case combinations of weathering plus high-shear wear to release free CNTs in the order of mg/m^2^/year. Synergy of chemical degradation and mechanical energy input is identified as the priority scenario of CNT release, but its lab simulation by combined methods is still far from real-world validation.

**Graphical Abstract:**

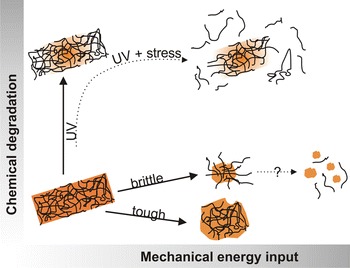

**Electronic supplementary material:**

The online version of this article (doi:10.1007/s11051-013-1504-x) contains supplementary material, which is available to authorized users.

## Introduction

**Fig. 1:**
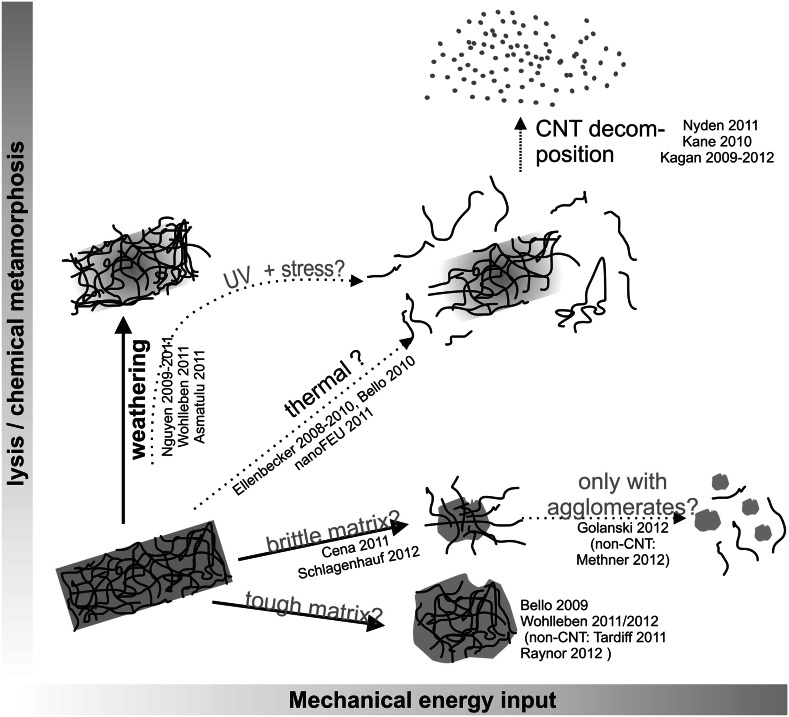
Emerging structure–property relationships for intermediate structures during degradation, starting from the intact nanocomposite (*bottom left*). *Thick arrows * point to degradation structures that have been observed before; mechanistic assignments in *blue* designate hypotheses to be tested in the present paper. Full references are discussed in the text and cited in the bibliography. (Color figure online)

All materials undergo life-cycle processes and ultimately lose their structural integrity, thus releasing solids that were originally embedded within that material. Nanocomposite materials consist of nanofillers embedded in a matrix of varying chemical composition, including for example cements and plastic polymers or polymer coatings. There are concerns that engineered nanomaterials, such as multi-wall carbon nanotubes (CNTs), may be released to humans or the environment from degradation of nanocomposite-enabled products, from degradation of waste from these products at the end of the life-cycle, and from waste materials generated during manufacturing. Matrices may be disrupted mechanically by machining and/or stress crack formation (Fig. 1, horizontal axis), or chemically by dissolution or shortening of polymer chain length from various processes, such as chemical or UV light exposure (Fig. 1, vertical axis). Nanocomposite materials with embedded nanofillers may hence release these nanofillers in certain scenarios of use and disposal (Nowack et al. 2012). If the nanofiller has been identified to represent a human health or environmental hazard the extent of polymer degradation and possible CNT release should be assessed before commercialization of a product that contains them to ensure that human and environmental exposures remain safe (Petersen et al. 2011; Som et al. 2010). Especially CNTs are a known hazard upon inhalation (Landsiedel et al. 2010; Ma-Hock et al. 2009), but only two dozen papers have reported experimental data on release from engineered nanofiller composites, even fewer on CNT composites (Kuhlbusch et al. 2011; Petersen et al. 2011).

The chemical metamorphosis by e.g., outdoor use of consumer articles (Fig. 1) first degrades the less persistent matrix, before CNTs are also decomposed. As the most consumer-relevant scenario, weathering degrades the matrix, leaving behind an entangled and collapsed network of CNTs (Fig. 1 “weathering”) (Nguyen et al. 2011; Wohlleben et al. 2013). Compared to the pure matrix, the CNT-polymer composite has been observed either to accelerate weathering for polyoxymethylene (POM) (Wohlleben et al. 2011) or on the contrary to form a passivation layer that slows down further weathering (for epoxy) (Asmatulu et al. 2011; Nguyen et al. 2011). While a release of nanoparticle fillers after weathering is expected in analogy to classical pigment coatings (Allen et al. 2004; Day 1990) and has been observed (Kaegi et al. 2008), it remains unknown by which treatment a weathered CNT-polymer composite will actually release free CNTs from their entangled, and partially still polymer-embedded network (Petersen et al. 2011). Here we propose a simple “UV + shaker” protocol to simulate release by outdoor use, and we characterize and quantify the resulting fragments by variations of the post-weathering shear input (Fig. 1 “UV + stress”).

Mechanical stresses include not only abrasion by normal consumer use, but also on a higher energy scale production processes such as sanding or shredding. What would we expect for these processes from classical fiber composite theory? Assuming a randomly oriented discontinuous CNT network and efficient interfacial stress transfer, the increase of the elastic modulus can be calculated and was found to be in good agreement for several CNT-epoxy composites (Lachman and Wagner 2010). In destructive testing such as sanding, cracks propagate through the composite material. Part of the energy is dissipated by fiber (CNT) pull-out against their matrix adhesion, characterized by τ_i_ the interfacial shear strength between CNT and matrix. The fibers (CNTs) absorb additional energy by breaking, if they surpass the critical length *l*_c_ defined as

$$ l_{\text{c}} = {{r_{\text{CNT}} \sigma_{\text{CNT}} } \mathord{\left/ {\vphantom {{r_{\text{CNT}} \sigma_{\text{CNT}} } {\tau_{\text{i}} }}} \right. \kern-0pt} {\tau_{\text{i}} }} $$

with *r*_CNT_ the radius, *σ*_CNT_ the tensile strength (with typical values of *r*_CNT_ = 15 nm and *σ*_CNT_ = 130 GPa) (Barber et al. 2005). The values of τ_i_ depend on the specific chemistry; for pristine and aminated CNTs in epoxy values between τ_i_ = 30 and 140 MPa were determined (Coleman et al. 2006). The resulting critical length *l*_c_ ranges between 7 and 65 μm, clearly above the average length *l*_CNT_ of a few μm. Hence, in contrast to conventional fillers, CNTs should not break but instead be pulled out, with the longest possible pull-out length equal to half the CNT length. From this perspective, CNTs should generally form protrusions at failure surfaces.

Among the few studies to characterize the fragments released from machining of CNT-epoxy (Bello et al. 2009; Cena and Peters 2011; Golanski et al. 2012; Huang et al. 2012), CNT-polyurethane (TPU) (Wohlleben et al. 2013), CNT-cement, or CNT–POM (Wohlleben et al. 2011) composites, there is so far a consensus that the debris mass is dominated by micron-sized composite fragments of matrix with bound CNTs, not by freely released CNTs, neither individually nor in bundles, as evidenced by electron microscopy of collected samples. The same applies to nanoplatelet composites (Raynor et al. 2012; Sachse et al. 2012a, b) and pigment composites with various polymers (Göhler et al. 2010; Koponen et al. 2009; Koponen et al. 2011; Saber et al. 2011; Vorbau et al. 2009). The surface of the CNT composite fragments, however, was in some cases decorated by a hairy layer of (presumably) CNTs protruding from the particle (Cena and Peters 2011; Schlagenhauf et al. 2012) and it is presently unknown whether these are indeed naked CNTs and whether this phenomenon is universal. Individual fillers were identified in filtered air samples after abrasion of polymer containing elevated CNT content (4 % by weight, Huang et al. 2012), CNT agglomerates (Golanski et al. 2012), and fibers that were intermediates between CNTs and conventional carbon fibers in terms of diameter, length, and stiffness (Methner et al. 2012). From all the above studies, quantitative values for the content of free CNTs in the debris powder are missing.

Here we provide comparative data on protrusions and develop a rationalization to predict the occurrence of protrusions (Fig. 1 “brittle”). For the first time, we establish quantitative upper limits on the amount of freely released CNTs from individual and combined scenarios of weathering and mechanical stresses (Fig. 1).

An important process that we do not address by experiments is the fast matrix degradation at increased temperatures. During dry core drilling (Bello et al. 2010; Sachse et al. 2012a) or during compounding, polymer vaporizes and re-condenses into airborne particles (polymer fumes), which may entrain particulate nanofillers (Ashter et al. 2010; Tsai et al. 2008), whereas this was not observed with CNTs as nanofiller (Fleury et al. 2011). Above the matrix decomposition temperature, degradation gases may impart enough mechanical energy to disrupt intact CNTs from their network as studied by the *nanofeu* project (Calogine et al. 2011; Motzkus et al. 2011). The thermal route is hence a diagonal in the scheme of Fig. 1. The CNTs themselves will burn around 600 °C (Mansfield et al. 2010), and were found to be absent in gases of well-ventilated combustion (Uddin and Nyden 2011), whereas metal-oxide nanofillers must and can be retained by standard air filters (Walser et al. 2012). Non-thermal degradation of CNTs was reported only for rather unique environmental or physiological conditions (Kagan et al. 2010; Liu et al. 2010).

## Materials and methods

### CNT-epoxy

The experimental setup for generating CNT-epoxy sanding particles has been described in detail by Huang et al. 2012. Briefly, test samples (12.5 × 1.3 × 0.5 cm) were prepared by mixing 2 % by weight multi-wall CNTs (10–50 nm outer diameter, 1–20 μm length; Baytubes, Bayer Material Science, LLC, Pittsburgh, PA) with epoxy resin. The mixture was poured into a mold and baked in an oven. The test samples were then removed from the mold and placed in an automated sanding simulation system equipped with sandpaper (Huang et al. 2012). The samples prepared for electron microscopy represent a portion of the material (~4 mg) residual from sanding either neat or 2 % CNT test sticks with 220-grit sandpaper (model 320240; 3M, St. Paul, MN), or 2 mg of bulk CNTs. The material was dispersed in 4 mL acetone, and the suspension was sonicated in an ultrasonic bath for 1 min. A portion of suspension was dispersed onto a lacey carbon substrate supported by a transmission electron microscopy grid with the Formvar layer removed (Model 01890, Ted Pella Inc., Redding, CA). Lacey carbon grids were selected because their open structure provides areas with no background interference for improved image quality.

Images of the samples and elemental information were obtained using an SEM/Scanning Transmission Electron Microscope (STEM, S-5500 ultrahigh resolution SEM with STEM capabilities, Hitachi High Technologies America Inc., Gaithersburg, MD) equipped with energy-dispersive X-ray spectroscopy (EDS) system (Bruker Quantax with XFlash silicon drift detector, Bruker AXS Inc., Billerica, MA). General surface morphology and chemical composition of a representative subset of collected airborne particles were examined. We analyzed by SEM more than a dozen samples collected from the sanding dust. Over one hundred individual particles were examined. An advantage of the SEM/STEM used in this study was the ability to obtain secondary electron and transmission electron images of the same relative area of a sample. Additionally, this microscope permitted toggling between dark-field and bright-field STEM to identify particles in the samples with high atomic number within their protrusions. These areas were then further analyzed through elemental maps to identify the presence of metal catalysts. Iron and nickel metal catalysts were reported by the CNTs manufacturer to be used during the production process.

### CNT-cement, CNT–TPU, and CNT–POM

The experimental setup has been described in detail by Wohlleben et al. (2011). Briefly, background particle concentrations are reduced to 250 particles/cm^3^ inside a chamber with a filtered air supply. The preparation of samples was described previously (Wohlleben et al. 2011). In short, the content of Nanocyl CNTs of type NC7000 (Nanocyl s.a., Sambreville, Belgium) was 2 wt% in cement and 3 wt% in TPU. The CNT content in CNT–POM is less than 5 wt%. A 10-cm-diameter specimen (2 mm or 3 mm thickness) rotates against sanding paper (specification KK114F with a grain size P320 from VSM, Hannover, Germany, abrasive material is aluminum oxide nominally) at a relative velocity of 6.5 m/s, at 2,000 rpm. Airborne fragments are aspirated onto a membrane filter and fragments fallen from the sample holder are collected for further investigation (SEM, SIMS, XPS, laser diffraction, AUC).

Weathering: UV radiation on conventional paints causes the release of pigments known as chalking. Standardized tests are established for coatings, and we primarily adhere to ISO 3892-2:2006 (with apparatus Suntest™ XLS+, standard-black temperature 65 °C), where only UV irradiation (111 W/m^2^ at 300–400 nm) without simulated rain is performed. We can thus ensure that degradation products are not blown or washed away, but can be safely detected and characterized. The application to nanocomposites has been described before (Wohlleben et al. 2011, 2013). We exposed the nanocomposite and the reference testing plates in parallel for 4 (and 8) weeks, equivalent to 9 (and 18) months in Europe at ~50° northern latitude, corresponding to an acceleration factor 8. For comparison, wet weathering was also performed, again using equipment standardized in ISO 4892-2 (Verf. A): Humidity cycle (102 min dry + 18 min rain), at average relative humidity of 50 ± 10 %. The wet weathering used lower intensity of 60 W/m^2^ (300–400 nm), but was kept longer (1243 h), so as to simulate the same 9-months treatment, at the same standard-black temperature of 65 °C. After either dry or wet weathering, the surfaces were characterized by SEM and XPS.

Release by weathering: UV + shaker method: The combined action of matrix degradation and mechanical stress on the remaining CNT network was assessed on thermoplastic polyurethane with 3 % CNTs as a first exploratory test case. To assess actual release from 12 samples, each weathered piece with surface area of 42 cm^2^ was immersed in a surfactant solution (2.5 ml H_2_O + 0.5 g/l SDS) and placed on a rather vigorous shaker for 24 h (5 Hz, 1 cm amplitude), then in an ultrasonic bath for 1 h, and then treated by ultrasonic probe for 30 s. At each step, the concentration of fragments released into the solution, among them possibly free CNTs, was assessed by interference-AUC (see below) and by SEM.

Photoelectron spectroscopy (XPS) was performed with a Phi XPS 5500 system with 300 W monochromatic Al-K alpha radiation, pass energy for surveys 117 eV (measurement time of 45 min), and detailed spectra at 23.5 eV (measurement time of 6 min). Evaluation performed by CasaXPS 2.3.15, based on the Phi standard-sensitivity factors, with Shirley background subtraction and peak shape fits as sum of 90 % Gaussian and 10 % Lorentzian. Information depth is limited to the surface 10 nm of the material. We measured three XPS replicates: Powders were gently pressed and measured on three locations. Each location integrates a sample area of 0.5 mm^2^, hence represents at least hundreds of fragments. The variance was found to be below the 1 at.% level.

The SEM measurements were performed with a JEOL JSM-7500 TFE SEM at an accelerating voltage of 5 kV. The sample surfaces were sputtered with an ~15-nm-thin Pt layer prior to the SEM imaging in order to prevent charging of the surfaces due to the electron beam.

### Quantification of released CNTs

**Fig. 2:**
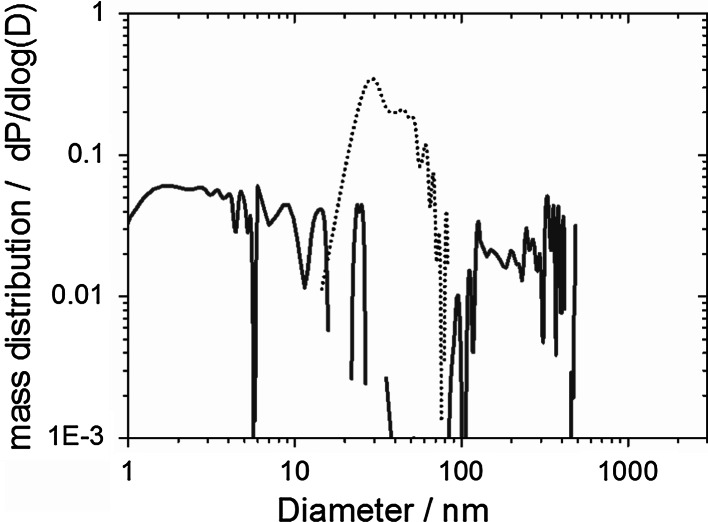
Positive and negative controls for the size distribution of suspended sanding fragments. Water as negative control (*blue solid line*); a CNT suspension as positive control was measured after the same de-agglomeration protocol (*black dotted line*). (Color figure online)

The detection equipment employed, known as analytical ultracentrifugation (AUC) (Carney et al. 2011; Planken and Colfen 2010), is especially suited to quantify traces of colloids within a heterogeneous mixture, as validated by deliberately mixed samples (Wohlleben 2012) and well established for CNT dispersions (Arnold et al. 2008). AUC quantifies the amount and the diameter of dispersed nanofillers and composite fragments independent of each other (0.5–10,000 nm diameter) (Planken and Colfen 2010). The AUC analysis uses at least 2 measurements for one data point, which represents about 10^9^ particles. Here we use interference optics (Beckman model “XLI proteome lab”) and the raw data are fitted by the freeware software SedFit. The mass concentrations read directly from the interference fringe shift without further conversion. The protein signal was evaluated with *ρ* = 1.36 g/cm^3^ and dn/dc = 0.18 cm^3^/g. To define the detection limit for degradation fragments in the sub-100-nm region, we measured a water blank with interference-AUC and obtained the curve shown in Fig. 2, blue line as negative control.

Assuming a typical refractive index increment dn/dc = 0.2 cm^3^/g, the integrated area under these curves gives a concentration of 0.05 mg/ml = 50 ppm. This noise level defines the detection limit. Note that interference optics are strictly linear with the concentration. No saturation occurs, and no Mie correction is required. The de-agglomeration protocol used probe ultrasonication (UP200S from Hielscher GmbH, used at 75 W, 2 min., 24 kHz, max. 600 W/cm^2^) with albumin, since several authors have reported that albumin is an effective dispersing agent for CNTs (Nepal and Geckeler 2007; Poland et al. 2008). We verify with our specific CNTs that the medium of suspension (RSA 25 mg/ml in water) is effective in dispersing these CNTs, suspended at 5 mg/ml by probe ultrasonication. The interference centrifuge finds a characteristic signal at 15–60 nm (Fig. 2, dotted line). The signal is significantly above the detection limit, and the hydrodynamic diameter is as expected for tubes closer to the cross section than to the length as measured by TEM on a pure CNT reference. This positive control confirms a good individualization by the above protocol.

## Results

### CNTs protruding from fragments after sanding: epoxy

Electron microscopy was performed to characterize the protrusions on the airborne particles released when sanding CNT-epoxy nanocomposites. First, the bulk CNTs used as the nanofillers were analyzed to identify signature features for later identification. Analysis of bulk CNTs revealed the presence of dark regions corresponding to high-atomic-number areas. Elemental maps revealed that the high-atomic-number areas were iron and nickel, residuals of the metal catalysts used in CNT production.

Next, we analyzed the collected airborne particles that were released from sanding CNT-epoxy nanocomposite material. These particles were typically larger than 1 μm and appeared irregular in shape. Several protrusions extended from the main core of the particles and were consistent in size (10–50 nm outer diameter) and shape of bulk CNTs. Although the electron microscopy analysis was not quantitative, these protrusions were observed on most particles analyzed and 5–7 protrusions could be counted on a typical STEM image of a 1-μm particle. Larger particles presented more protrusions. STEM images, however, represent two-dimensional images of a particle’s outline; therefore, only those protrusions parallel to the field of view become visible. Protrusions extending toward or away from the viewer are not distinguishable in these images but are expected to exist in similar quantities. Neat epoxy particles were also larger than 1 μm and irregular in shape; however, no protrusions were found on their surface.

**Fig. 3:**
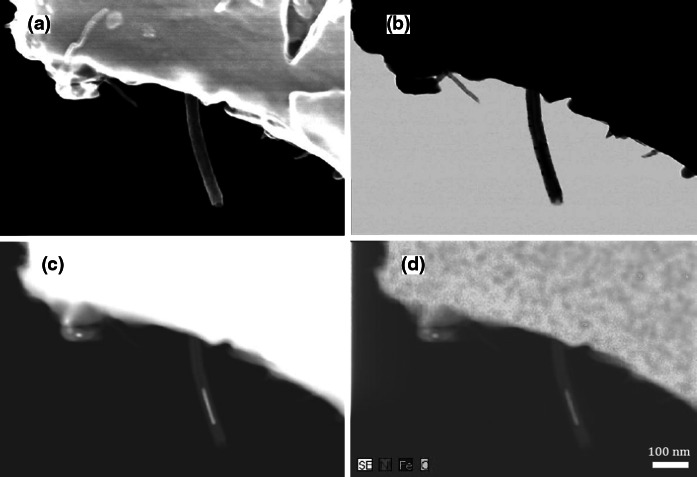
Secondary electron **a**, bright-field STEM **b**, dark-field STEM **c** images, and elemental mapping **d** of the protrusions of a MWCNT-epoxy nanocomposite particle

Images of the particles from sanding CNT-epoxy nanocomposite obtained at higher magnification and different imaging modes in the SEM/STEM confirmed that the protrusions are CNTs (Fig. 3). The morphology of the protrusions that extended from the main core of a particle (secondary electron image in Fig. 3a; and bright-field STEM image in Fig. 3b) was consistent with the tubular shape and OD of bulk CNTs.

Further investigation by dark-field STEM (Fig. 3c) revealed the areas of high atomic number within some of the protrusions that were confirmed to be composed of iron (blue) and nickel (red) through elemental mapping (Fig. 3d). This indicates that the protrusions are exposed CNTs.

### CNTs protruding from fragments after sanding: other materials

**Fig. 4:**
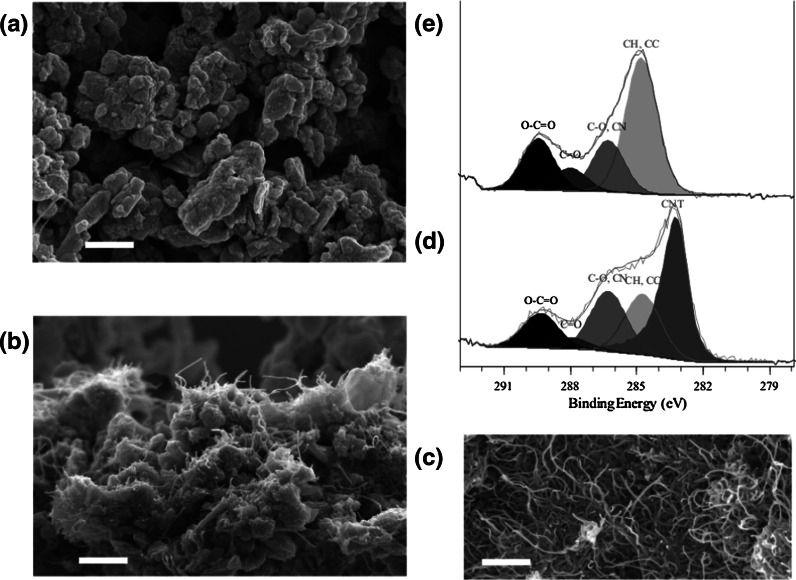
Protrusions on sanding fragments of a CNT-cement composite. Representative SEM scans with 500 nm *scale bars*: **a** cement negative control fragments; **b** CNT-cement composite fragments; **c** CNT positive control on the same scale. Chemical identification by XPS *line shape* analysis of the photoelectrons emitted from the surface-exposed C-atoms identifies 30 % of carbon as CNTs in the CNT-cement composite fragments (**d)**, while only organic additives were present in the cement reference sample (**e**)

**Fig. 5:**
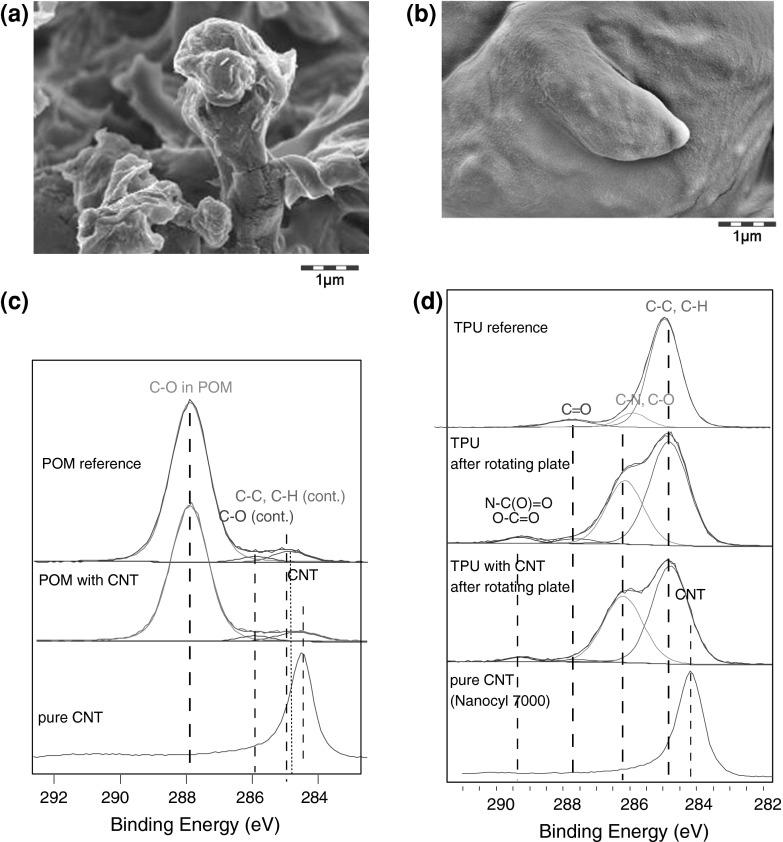
Sanding fragments from **a** + **c** CNT–POM composites and **b** + **d** CNT–PU composites, showing no protrusions, as demonstrated by their morphology in SEM (**a**, **b**) and their photoelectron spectra from XPS (**c**, **d**), benchmarked on the positive control of pure CNTs with *curve shape* fits to known carbon binding states. The *vertical lines* are guides to the eye for ease of comparison between spectral components

Here we present a detailed analysis of the particles recovered from sanding cement with embedded CNTs (Fig. 4a, b). The morphology of the protrusions extending from CNT-cement sanding particles was consistent with the shape of bulk CNTs (Wohlleben et al. 2011). Protrusions are clearly visible (Fig. 4b) and match the morphology and diameter of naked CNTs (Fig. 4c). A detailed line shape analysis of the 1-s photoelectrons from Carbon atoms reveals their coordination chemistry and shows a distinct signal at 283.2 eV that is characteristic for these CNTs (Fig. 4d, see Fig. 5 for a CNT positive control). In contrast, this signal is absent in the powder recovered from cement without CNTs (Fig. 4e). The chemical characterization indicates that the CNTs are not only embedded in the cement sample, but are also to a significant extent exposed on the accessible fragment surface.

The SEM micrograph does show CNTs, but none of the visible CNTs have both ends free. All visible CNTs emerge from cement grain surfaces, with one end of the tube still embedded in the inorganic matrix, and a loose end forming a “hairy layer” with length around 0.3 μm. This interpretation would match the actual application purpose of CNTs that are added to reinforce the mechanical strength *between* cement grains at the preferred failure interfaces.

To compare with other polymer matrices, identical techniques were employed on the sanding fragments released from CNT to TPU (original data, same material as in Ref. (Wohlleben et al. 2013)) and from CNT to POM (original data, same material as in Ref. (Wohlleben et al. 2011)) and presented in Fig. 5. The absence of protrusions is confirmed by SEM morphology (Fig. 5a, b) and by XPS chemical identification (Fig. 5c, d). For both polymers, the line shape of the Carbon 1 s photoelectrons, which reflects the chemical bonds, is identical whether or not the nanocomposite contains CNTs and for the reference polymer without CNTs. The characteristic shoulder of CNT photoelectrons (Fig. 5c, bottom line) that was dominating the surface for the CNT-cement (Fig. 4d) can be excluded. This observation is visualized by the line shape from CNT to TPU (Fig. 5d).

For CNT–POM, the small contribution from aliphatic C–C and C–H groups, which is shifted only 0.3 eV from the C–C–C–… in CNTs, is close and partially overlapping. However, a curve fit results in 0.8 % contribution of CNTs from the reference (where they are not present) and for the nanocomposite alike. We have hence identified two very different plastic polymers without notable protrusions of CNTs after sanding.

Correlation of the size of the hybrid fragments to materials and apparatus parameters goes beyond the scope of the present contribution.

### Are CNTs released by mechanical degradation alone?

**Fig. 6:**
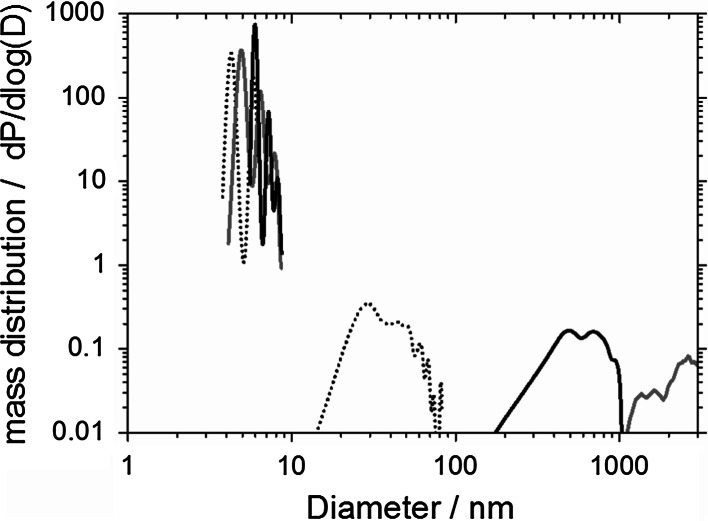
Low-diameter tail of the size distribution of suspended and de-agglomerated sanding fragments of nanocomposites. The as-prepared suspensions of CNT-cement (*black line*) and CNT–POM (*red line*) fragments, compared against the positive control (*black dotted line*). (Color figure online)

We suspended the powder from CNT-cement sanding in the same medium with the same protocol as for the positive and negative controls described in the methods section and obtained the size distributions shown with black solid lines in Fig. 6.

The integrated fringe shift quantifies that 3 wt% of the suspended 50 mg/ml is dispersed in the sub-micron range. The majority of the particles have significantly larger diameters between 1 and 20 μm, as measured by laser diffraction in excellent accord with the SEM images and with the centrifuge measurement of the low-diameter tail (Fig. 6).

Most importantly, the centrifuge signal in the characteristic size range below 100 nm, where free CNTs would appear is not distinguishable from the detection limit (50 ppm, see above) in this specific suspension with 50,000 ppm solid content. The ratio establishes an upper limit of 0.1 wt% of the original nanocomposite material released into the characteristic size range below 150 nm, where free CNTs would contribute. Compared to the original CNT content, at least 95 % of the CNTs remain embedded in the cement matrix (including those CNTs that protrude onto the surface). Along the same rationale, at least 95 % of the CNTs remain embedded in POM matrix and are not released (red line in Fig. 6). The actual value could be 100 %, but cannot be better quantified with current methods. In the PU composite fragments, we detected signals around 0.2 wt% (just above the limit of detection) in the range below 100 nm where free CNTs would appear, but the same content in this range is found for the reference material without CNTs (Wohlleben et al. 2013).

The recurring colloidal signal at 5 nm can be understood by converting it to a molar mass, giving peaks at 75 kDa and weaker at 150 kDa, identified as RSA monomer and dimer with slight deviation from literature attributed to viscosity mismatch. From the integrated fringe shift of these specific peaks, we know that 85 % of the RSA is not adsorbed, (Schaefer et al. 2012) and further tests by Secondary Ion Mass Spectroscopy (SIMS) confirmed that the remainder of the RSA is adsorbed on the sanding powders (Wohlleben et al. 2011). The proven accuracy even below 10 nm further adds to the validation of the absence of signal in the CNT-specific diameter range.

### Fragments released after UV weathering + mechanical stress

After either dry or wet weathering, the surfaces were immersed in a surfactant solution and placed on a shaker for 24 h, then in an ultrasonic bath, and then treated by ultrasonic probe. Prior to treating the solution with the shaker and ultrasonic agitation, no color change or turbidity was observed. After the shaker and any of the ultrasonic treatments, the suspension medium turned turbid. The colors of the suspensions, yellowish from TPU and gray/black from TPU + CNT, indicated the presence of either free CNTs or CNTs bound within small polymer fragments.

**Fig. 7:**
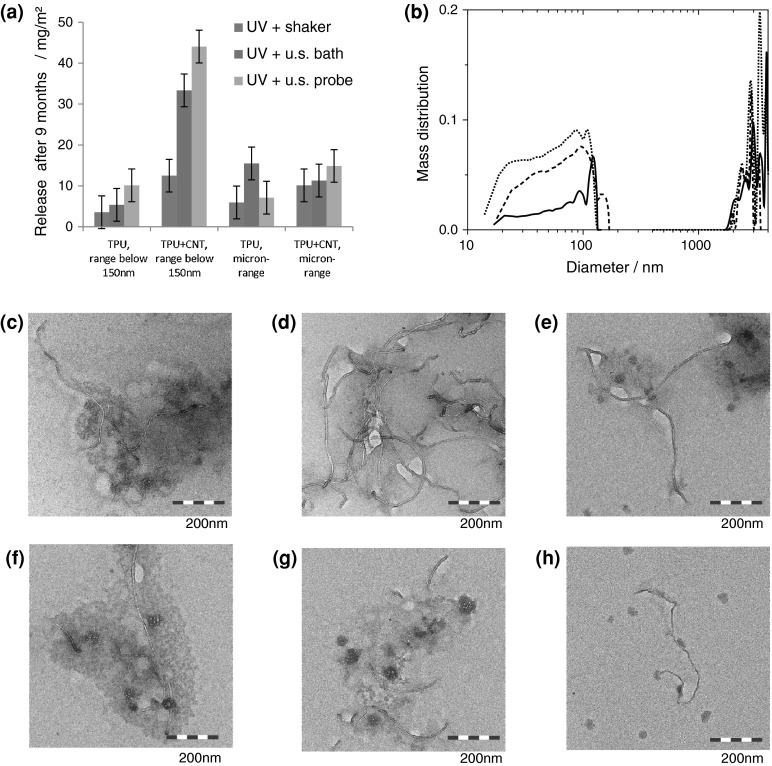
Release by the combined action of weathering and mechanical methods, on the example of a TPU + CNT composite. **a** Summary in units of released mass per irradiated surface for both TPU–CNT and TPU materials with the uncertainty of the detection method indicated; **b** size-selective quantification of release on the example of TPU–CNT after UV weathering (*solid*: +shaker; *dashed*: +ultrasonic (u.s.) bath; *dotted*: +ultrasonic probe). Characteristic TEM images from several coexisting morphologies of released material after UV-only weathering and **c** shaker, **d** u.s. bath, **e** u.s. probe. Analogously, TEM images after UV + rain weathering and **f** shaker, **g** u.s. bath, **h** u.s. probe. A gallery of coexisting morphologies and sizes on 50 times larger scan areas is presented in the Supporting Information (Fig. SI_1)

By size characterization of all samples, both micron-sized fragments and fragments smaller than 150 nm in diameter were observed. Free CNTs, if present, would be found among the fragments smaller than 150 nm. Such small fragments are observed for all samples, including the weathered reference samples without CNTs, but to a varying degree (Fig. 7a). To indicate the order of magnitude, the observed colloidal content is indicated in units of mg release per m^2^ composite surface per year-equivalent irradiation.

The most striking observation is the very strong dependence of released quantities on the arbitrarily chosen mechanical energy input, especially for the CNT-containing composite (Fig. 7a). In SEM images of dried suspensions, hybrid μm-sized fragments of polymer matrix with embedded CNTs coexist with smaller polymer-CNT fragments and with occasional free CNTs (Fig. SI_1, Supporting Information). With higher mechanical energy input, the share of the smaller fragments increases (Fig. 7c–e), thus confirming the size characterization by AUC. If the technically highest possible shear forces are employed, free CNTs can be observed (Fig. 7e, h). However, their concentration in the thus-simulated run-off waters depends strongly on the shear forces, as demonstrated by the solid/dashed/dotted lines in Fig. 7b, and they are always mixed with smaller and larger hybrid polymer-CNT fragments. The same phenomena are observed for humidity cycle weathering (Fig. 7f–h), where again the share of smaller fragments and occasional free CNTs can be increased by a deliberate choice of the applied mechanical energy.

The release of micron-scale fragments is not sensitive to the mechanical energy input, and is quite independent of the presence of CNTs in the matrix. The release in the range below 150 nm is around 12 mg/m^2^ after 9 months of dry weathering by the prolonged (24 h) shaking procedure. In comparison, the background levels were less than 5 mg/m^2^ for the matrix without filler. Lack of validation of the proposed methods, however, makes it difficult to relate these numbers to actual release scenarios.

**Fig. 8:**
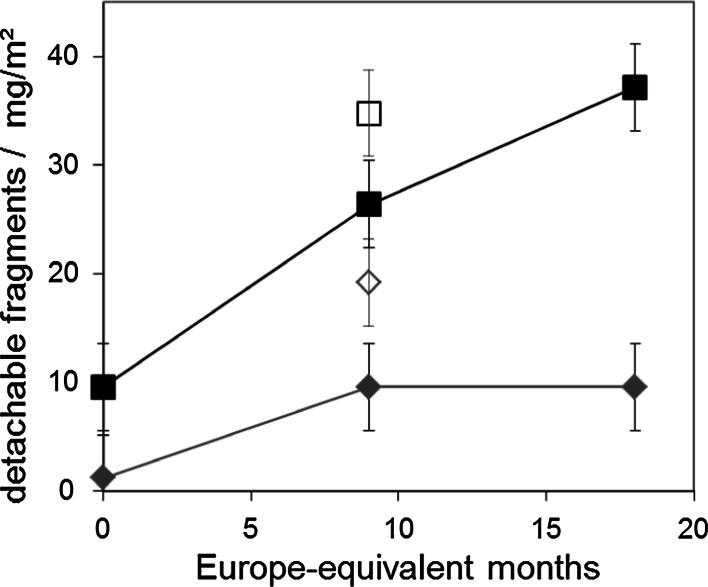
Time course of release after weathering equivalent to central European latitudes with rain (*open symbols*) and without rain (*filled symbols*). The detachable fragments from TPU + CNT (*blue*) increases over time both. Also, from the reference TPU (*red*) some fragments can be detached, but significantly less. (Color figure online)

In an independently repeated experiment, we assessed the time course of release after weathering. The degradation is progressive with time, but seemingly less than linear (Fig. 8). However, this effect is also observed for the matrix without CNTs, and hence cannot be attributed to passivation effects by the CNTs that remain on the surface. Note that the time scale and order of magnitude of release may already be different for other polyurethanes (e.g., with polyester-based instead of polyether-based) and for other polymer matrices.

## Discussion

Our results demonstrate that protrusions after mechanical stress are not universal, but occur on seemingly unrelated materials, including epoxy (Cena and Peters 2011; Schlagenhauf et al. 2012) and cement (Wohlleben et al. 2011). Epoxy is a polymer matrix of high tensile strength, where protrusions were already observed years ago in an unpublished study by the Batelle Laboratories. In contrast, cement is an inorganic matrix of high compression strength, but low tensile strength and completely different interfacial binding of CNTs. Contrary to the expectations from pull-out theory, we found no indications of protrusions by either photoelectron shapes, catalyst tracer elements (XPS, SIMS), or by morphology (SEM) on the two thermoplastic CNT composites, the Polyoxymethylene (Wohlleben et al. 2011) and the Polyurethane (Wohlleben et al. 2013). To rationalize the contrasting findings, Table 1 assembles the relevant tensile and thermal properties.

**Table 1:** Thermal and mechanical properties of matrix materials showing CNT protrusions or not after sanding

Matrix	Testing temperature	Tensile modulus/Gpa	Ultimate tensile strength/Mpa	Elongation at break/%	Protrusions	References
None (pure mwCNT)		8,000	>11.000	–		(Yu et al. 2000)
Cement	Below *T*_decomp_. (no *T*_g_, no *T*_melt_)	11	2	1	Yes	(Odelson et al. 2007; Wohlleben et al. 2011), here
Epoxy	Below *T*_g_, below *T*_decomp_ (no *T*_melt_)	3.5	69	5	Yes	(Cena and Peters 2011; Schlagenhauf et al. 2012) here
POM	Above *T*_g_, below *T*_melt_	2.5	65	27	No	(Wohlleben et al. 2011)
TPU	Above *T*_g_, below *T*_melt_	0.02	45	600	No	(Pattanayak and Jana 2005; Wohlleben et al. 2013)

The correlation of the protrusion phenomenon is virtually zero with ultimate tensile strength, and a threshold in tensile modulus is not evident: Epoxy and POM have very similar tensile strength, but only epoxy shows protrusions. Cement and epoxy differ significantly in tensile modulus and strength, but both show protrusions. Some of the materials are visco-elastic, others entropy-elastic, and others energy-elastic. However, the protrusions correlate nicely with an elongation at break of only a few percent for the matrix material. We propose that the polymer, elongating more than 10 % and forming necks does not allow CNTs to emerge on a purely geometric basis. Note that all materials are tested far below the melt or decomposition temperature. Indeed due to necking some newly formed polymer fibers can be observed in TPU sanding fragments (Wohlleben et al. 2013). Note that the elongation parameter is in general compatible with the fracture toughness parameter proposed by Schlagenhauf et al. (2012).

We conclude that protrusions are not necessarily progressive with energy input, but a material-dependent phenomenon in mechanical degradation (Fig. 1 scenario “brittle vs. tough”). Based on the correlation to the elongation at break, one would expect protrusions to occur on polystyrene, polyimide, and polyacrylic CNT composite fragments (all below 10 % elongation at break). In highly filled polymers, e.g., synergistic filling with carbon fibers and CNTs (Sager et al. 2009), the much reduced elongation at break is even more likely to allow for CNT protrusions on fragments. In the majority of thermoplastics with elongation at break around 100 % we expect only minimal occurrence of protrusions after sanding: polyamide, polyethylene, polycarbonate, PET, and ABS. Future work should involve quantitative microscopy to assess the frequency of protrusions on polymer fragments. It remains to be tested whether the rules derived from sanding apply also for cutting and shredding, where knifes could restrict the polymer necking that we believe to embed the CNTs.

The intriguing question whether protrusion structures necessarily induce a physiological response has been investigated elsewhere and the in vivo lung instillation found no additional inflammation compared to the same cement without CNTs (Wohlleben et al. 2011). The same resulted for TiO_2_ embedded in paints (Saber et al. 2011, 2012). The additional in-depth investigations reported here on aliquots of the original CNT composite material confirm that these in vivo results apply to nanocomposite fragments with protrusions.

Further, we explored a complementary method for size-selective quantification of freely released CNTs after suitable dispersion protocols. Our measured value of at least 95 % of the CNTs remaining embedded is in perfect agreement with the visual evaluation, and adds the first quantitative benchmark. We found no positive indication of the presence of free CNTs, but at most 5 % (of the 2 % CNTs in the composite) may linger below our limit of detection (Fig. 1 scenario “only with agglomerates”). With a 100-fold improvement in the limit of detection provided by the Spin Analytical centrifuges with interference/Schlieren optics, we foresee a rapid progress in the quantification of free CNTs. Like the majority of suspension detection methods, the present results are mass-based, and cannot be compared directly to the number-based airborne particle distributions from the established aerosol methods. A significant advancement of number-based suspension methods is required to close this gap.

Regarding weathering, the hypothesis of Nguyen was developed on CNT-epoxy systems and states that the matrix is hydrolyzed from the top few hundred nm, leaving behind a network of CNTs that acts as a passivating and UV-protecting layer (Nguyen et al. 2011). On a structural level, we have confirmed earlier that such a CNT network does remain, and collapses due to van-der-Waals interactions to a dense layer on the receding polymer (see also Fig. 1 scenario “weathering”) (Wohlleben et al. 2013). Concerning the passivating function, we used lower UV intensity (8 times instead of 22 times acceleration), which were much less sensitive to weight loss due to thick samples instead of thin films. Scatter was too high to allow conclusions on this proposed passivating effect. From the data on POM (Wohlleben et al. 2011) and TPU (Wohlleben et al. 2013) composites with CNTs, we know that the matrix hydrolysis proceeds up to several μm deep. For POM a passivating effect could be ruled out, but POM is known to be photolabile in itself. In the contrary, there is evidence to support Nguyen’s hypothesis from CNT and graphene coatings that performed as efficient UV protectors that reduce weight loss and reduce contact angle changes (Asmatulu et al. 2011). Our novel studies show that only the combined action of weathering and mechanical stress induces release of fragments (Fig. 1 scenario “UV + stress”), whereas the absence of stress after weathering prevents the fragment’s release (Nguyen et al. 2011). Release of free CNTs can occur, but requires much more secondary energy input than the spontaneous release of pigments or nanoparticulate fillers, which were found already in run-off waters (Kaegi et al. 2008). In contrast, extreme shear by ultrasounds after UV irradiation was required to disrupt CNTs from their collapsed and entangled network. Our UV + shaker approach cannot be regarded as validated. The results demonstrate methodical gaps and may only serve as orientation for the observable phenomena.

## Conclusion

We simulated scenarios of high-energy input (sanding) and of known degradation to the polymer matrix (dry and wet weathering) to investigate the airborne particles released from polymer nanocomposites when they undergo potential life-cycle events. Bright- and dark-field STEM images, elemental mapping, and photoelectron line shape analysis have clearly identified protrusions of CNTs from fragments of CNT-epoxy and CNT-cement composites after sanding, whereas such protrusions were not observed for CNT-polyoxymethylene and CNT-polyurethane fragments. We proposed that the microscopic flow of polymers with an elongation at break above a few percent covered the pulled-out CNTs during the shear-induced separation of fragments.

We have no indication of freely released CNTs from mechanical forces alone. Based on size characterization with validated methods, at least 95 wt% of the CNT nanofillers remain embedded. Advancement of colloidal sizing and counting methods will push the limits of detection further.

In contrast, weathering can degrade the polymer matrix, such that protruding networks of the more persistent CNT nanofillers are uncovered. Whether this protruding layer has a passivating effect on further matrix degradation could not be identified unambiguously. Our orientating experiments indicate a release in the order of mg fragments per m^2^ surface per year-equivalent irradiation—for polymers without UV stabilization. Simulated run-off waters contain both micron-sized and smaller polymer-CNT fragments, and may also contain free CNTs. However, the deliberate application of very high shear forces on weathered surfaces is required to clearly observe free CNTs. A major effort in method validation and testing of UV-stabilized nanocomposites is required before any conclusions on the real-world release by weathering can be drawn.

## Electronic supplementary material

Below is the link to the electronic supplementary material.

Supplementary material 1 (PDF 281 kb)
