# Supplementary material for: Scenarios and methods that induce protruding or released CNTs after degradation of nanocomposite materials
Source: J Nanopart Res. 2013 Mar 6;15(4):1504. doi: 10.1007/s11051-013-1504-x (PMC3625415; doi:10.1007/s11051-013-1504-x)
Supplement: Supplementary file 1 — Supplementary material 1 (PDF 281 kb) [file 11051_2013_1504_MOESM1_ESM.pdf]

Scenarios and methods that induce protruding or released CNTs after degradation of nanocomposite materials

- Online Supporting Information

Sabine Hirth[1], Lorenzo Cena[2], Gerhard Cox[1], Željko Tomović[3], Thomas Peters*[4], Wendel Wohlleben*[1]

[1] *BASF SE, 67056 Ludwigshafen, Germany*

Tel +49 621 60 95339

Email: *wendel.wohlleben@basf.com*

[2] *CDC/NIOSH, Morgantown, WV 26505 USA*

[3] *BASF Polyurethanes GmbH, GMU/UE, Elastogranstrasse 60, 49448 Lemfoerde, Germany*

[4] *The University of Iowa, Iowa City, IA 52242 USA*

Email: *thomas-m-peters@uiowa.edu*

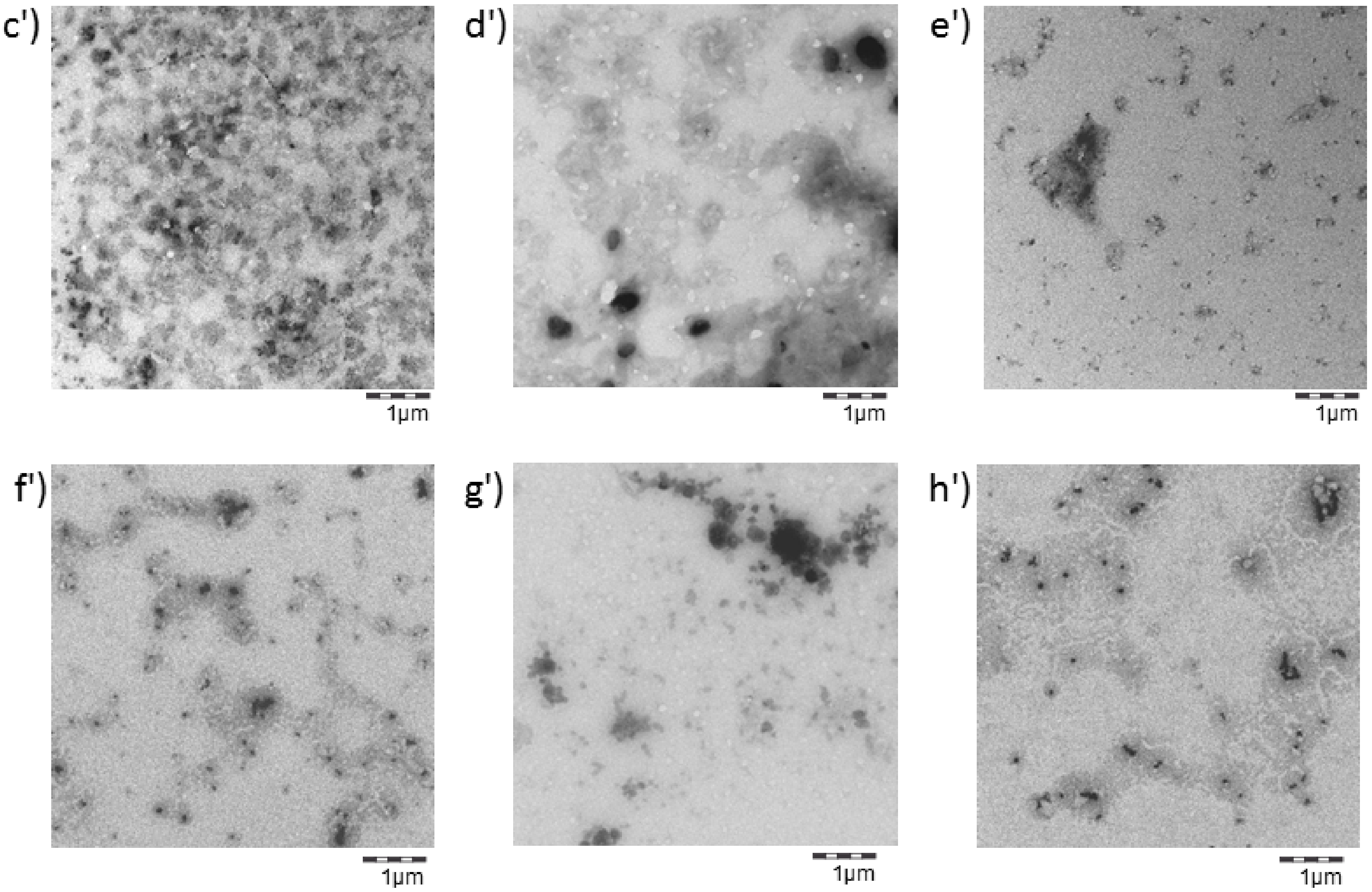

Fig. SI_1 Overview of the release by the combined action of weathering and mechanical methods, on the example of a TPU+CNT composite. Higher resolution TEM scans of characteristic individual fragments are shown in Fig. 7 c) – h) in the main text. Here we provide unfractionated TEM scans of the polydisperse and polymorph fragments of released material after UV-only weathering with c’) shaker, d’) ultrasound bath, e’) ultrasound probe. Analogously, TEM images after UV+rain weathering and f’) shaker, g’) ultrasound bath, h’) ultrasound probe.
